# Prognostic Significance of Lung Ultrasound for Heart Failure Patient Management in Primary Care: A Systematic Review

**DOI:** 10.3390/jcm13092460

**Published:** 2024-04-23

**Authors:** Anna Panisello-Tafalla, Marcos Haro-Montoya, Rosa Caballol-Angelats, Maylin Montelongo-Sol, Yoenia Rodriguez-Carralero, Jorgina Lucas-Noll, Josep Lluis Clua-Espuny

**Affiliations:** 1 Institut Català de la Salut (ICS), SAP Terres de l’Ebre, Primary Care Health Tortosa-est, 43500 Tortosa, Spain; 2 Programa Doctorado Biomedicines, University Rovira-Virgili, Campus Terres de l’Ebre, 43500 Tortosa, Spain; 3 Institut Català de la Salut (ICS), SAP Terres de l’Ebre, Unitat Docent Terres de l’Ebre-Tortosa, Primary Health Care Tortosa-est, 43500 Tortosa, Spain; mharo.ebre.ics@gencat.cat (M.H.-M.); mmontelongo.ebre.ics@gencat.cat (M.M.-S.); yrodriguezca.ebre.ics@gencat.cat (Y.R.-C.); 4 Institut Català de la Salut (ICS), SAP Terres de l’Ebre, Family and Community Medicine Unit in Primary Care Health Tortosa-est, 43500 Tortosa, Spain; rcaballol.ebre.ics@gencat.cat; 5 Regió Sanitària Terres de l’Ebre, CatSalut, 43500 Tortosa, Spain; jlucasn@catsalut.cat; 6 Fundació Institut Universitari per a la Recerca a l’Atenció Primària de Salut Jordi Gol i Gurina (IDIAPJGol), SAP Terres de l’Ebre, Institut Català de la Salut, 43500 Tortosa, Spain

**Keywords:** lung ultrasound, heart failure, B-lines, prognosis, mortality, hospital admissions, primary care

## Abstract

**Background:** Heart failure (HF) affects around 60 million individuals worldwide. The primary aim of this study was to evaluate the efficacy of lung ultrasound (LUS) in managing HF with the goal of reducing hospital readmission rates. **Methods:** A systematic search was conducted on PubMed, Embase, Google Scholar, Web of Science, and Scopus, covering clinical trials, meta-analyses, systematic reviews, and original articles published between 1 January 2019 and 31 December 2023, focusing on LUS for HF assessment in out-patient settings. There is a potential for bias as the effectiveness of interventions may vary depending on the individuals administering them. **Results:** The PRISMA method synthesized the findings. Out of 873 articles identified, 33 were selected: 19 articles focused on prognostic assessment of HF, 11 centred on multimodal diagnostic assessments, and two addressed therapeutic guidance for HF diagnosis. LUS demonstrates advantages in detecting subclinical congestion, which holds prognostic significance for readmission and mortality during out-patient follow-up post-hospital-discharge, especially in complex scenarios, but there is a lack of standardization. **Conclusions:** there are considerable uncertainties in their interpretation and monitoring changes. The need for an updated international consensus on the use of LUS seems obvious.

## 1. Introduction

The demographic evolution in developed countries is characterized by the emergence of new and complex health needs due to chronic multimorbidity. One of the conditions with the greatest impact is heart failure (HF) [1]. Currently, it affects about 60 million people worldwide, making it one of the chronic conditions with the greatest health and economic impact [2,3]. There is a great variability in epidemiological studies, but its prevalence increases with age being higher than 10% in patients over 70 years old and >14% in those over 75 years old [4,5,6] with an average incidence of 2–6 cases per 1000 inhabitants. In Spain, the estimated hospitalization rate is 2.37, with an average stay of 8.5 days and accounts for between 3% and 5% of hospital admissions [7]. The 30-day readmission rate is 20% [1], remaining the leading cause of hospitalization among patients ≥ 65 years old, and the third cause of cardiovascular mortality accounting up to 20%. Also, 55–70% of patients die within 5 years of diagnosis with a mortality rate of 20% per year, 50% at 5 years, and up to 80% at 10 years [8,9,10,11,12,13,14,15,16,17,18,19]. Each new hospitalization increases the mortality risk of these patients by 20%, so avoiding severe decompensation is considered crucial. Hospitalization is the inevitable consequence of this decompensation. Finally, around 40% patients are discharged from HF hospitalization prematurely when they are not ready to be discharged [20].

Studies predict that hospital admissions for heart failure will increase by 50% in the next 25 years [21] with re-hospitalization rates of up to 50% per year. Each hospitalization is a step backwards in the quality of life of patients with heart failure, and no cost-efficient solutions exist that can adequately screen, diagnose and monitor HF. The survival rate for heart failure has plateaued in the last 7 years, suggesting that additional measures are needed in addition to pharmacological treatments [2]. New therapeutic targets have allowed for the modifying of the natural history of heart failure in the last three decades, but mortality rates and recurrent hospitalizations remain very high in patients with HF. Despite these numbers, heart failure is still a relatively unknown disease, with a range of symptoms that many affected individuals initially attribute to other causes, delaying their diagnosis and treatment and, with it, their prognosis.

Since 1997 [22], lung ultrasound (LUS) technology has been widely utilized for assessing pulmonary congestion of cardiac origin. This is often indicated by the presence of B-lines, vertical artefacts that appear on ultrasound imaging of the lungs. These lines were associated with interstitial syndrome, a condition characterized by fluid in the interstitial spaces of the lung, and the researchers started recognizing the significance of B-lines in the context of heart failure. 

The knowledge and utilization of LUS in primary care are expanding as a clinical support tool for diagnosing and monitoring heart failure. This growth is based on published findings related to the outcomes of implementing a transitional care program between hospitals and primary care [23,24]; LUS is described as a rapid, mobile, and non-invasive method to monitor dynamic changes in pulmonary congestion, which may identify those at high risk for adverse events [25,26,27,28] more accurately than physical examination and lung X-ray [29,30]; additionally, it adds discriminative value to neuropeptides for the diagnosis, prognosis, and treatment of patients with decompensated HF with the use of a new CA19 biomarker [31,32,33,34]. Despite the evidence, there is currently no systematic implementation of LUS for standard monitoring of HF patients in primary care.

Most studies make no distinction between HF with preserved ejection fraction (HFpEF) and reduced ejection fraction (HFrEF) subtypes, despite the fact that HFpEF represents nearly half of all heart failure admissions [35,36,37,38,39], though it is clear that the HFpEF occurs predominantly in older females and patients with more comorbidities [40,41]. A coordinated care process from hospital discharge is essential to prevent decompensation and hospital readmissions: accessibility (quick contact to resolve any doubts or report any complications), longitudinality (clinical history and synergistic coordination with hospital care units), and comprehensiveness (ultrasound that improves resolution capacity, medication adjustment, and the need for re-evaluation) are three pillars of this review. Therefore, appropriate monitoring would lead to a decrease in readmissions, costs, and mortality. Providers are incentivized to use the model to identify high-risk patients, as it allows them to intervene early and potentially prevent heart failure exacerbations [14].

This study aimed to conduct a systematic review on the impact of integrating Lung Ultrasound in the management of heart failure patients, stratified by risk, with a focus on mortality and readmissions. Currently, there is a lack of evidence regarding: 1/the efficacy of B-lines as a determinant for hospital discharge in patients admitted for decompensated heart failure or in ambulatory follow-up settings; 2/the prognostic significance of B-lines when combined with other commonly utilized biomarkers and risk assessment tools in monitoring HF patients; 3/the existence of indications or guidelines for employing the predictive value of B-lines to guide out-patient therapeutic interventions during episodes of HF decompensation. The hypothesis is that LUS during follow-up in primary care will reduce the combined risk of worsening heart failure and/or cardiovascular death as well as symptoms and functional status in patients with heart failure regardless of their ventricular function.

## 2. Methods and Analysis

Host Organization: The Foundation University Institute for Primary Health Care Research Jordi Gol i Gurina (IDIAPJGol). Ethics Committee number 22/143-P.

Faculty or Research Center: University Institute for Primary Health Care Research Jordi Gol i Gurina (IDIAPJGol) and Ebrictus Research Group.

This systematic review was performed in accordance to the PRISMA (Preferred Reporting Items for Systematic Reviews and Meta-Analyses) guidelines

### 2.1. Search Strategy

A systematic search was conducted for scientific articles on PubMed, Embase, Google Scholar, Web of Science, and Scopus, including clinical trials, randomized controlled trials, meta-analysis, systematic reviews, and original articles that were related to the prognostic value of lung ultrasound in patients with HF from 1 January 2019 to 31 December 2023. The Boolean operator AND was used to combine the keywords and narrow down the search. Keywords include the following: Prognostic scores/Congestive heart failure/Cardiac insufficiency/Heart decompensation/Heart Failure/Pulmonary Congestion/Lung ultrasound/B-lines/Pulmonary echocardiography/Transthoracic echocardiography/ cardiopulmonary ultrasound/Echocardiographic assessment of the lungs/Lung diagnostic imaging/Ultrasonography Lung/Transitional care/Readmissions/Mortality.

**Figure 1:**
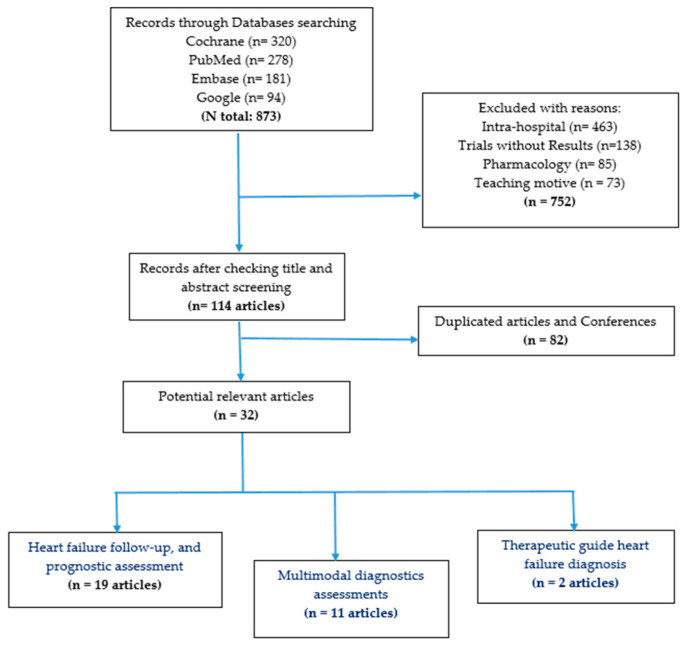
Results searching strategy.

Furthermore, we manually searched for relevant studies in a reference list of potentially eligible publications. The search in the literature was performed in February 2024. The articles were selected in two steps. In the first, the abstracts were checked and those not meeting the inclusion criteria were excluded. In the second step, the studies selected based on their abstracts were fully read, and those not meeting the inclusion criteria were excluded, according to the PRISMA model (Figure 1). Two researchers independently conducted a comprehensive search of biomedical literature databases, using a combination of subject terms and free words as well as snowball methodology.

### 2.2. Inclusion and Exclusion Criteria

All qualifying studies should meet the following criteria: articles approaching LUS managed by physicians for the assessment of congestion in patients with HF, followed up on an out-patient basis at their primary care center, and those discharged from hospital for new HF diagnosis or/and decompensation with preserved heart failure.

The gold standard for inclusion was the clinical diagnosis of heart failure by cardiologists or experienced physicians combined with history and relevant clinical examination; the language of the included literature was English; there is no restriction on the type of ultrasound instrument and the method of zonal lung ultrasound scanning, and the location of pulmonary ultrasound examination. The studies were excluded if they met the following criteria: duplicate publications; conference reports, editorials, letters, and case reports; studies with a sample size of <30 cases, severely reduced ejection fraction (LVEF ≤ 35%). The LUS may be employed in various scenarios, including emergency situations, as a treatment guide for in-patients, during hospital discharge, and for out-patient follow-up, but the studies using LUS in hospitalized participants were excluded except when was used around 72 h from hospital discharge and ambulatory follow-up.

Results were organized according to the management process in heart failure: risk/early-diagnosis-/follow-up/multimodal assessments/therapeutic guide:

(1) LUS accuracy in Heart failure diagnosis in out-patient settings, follow-up, and risk scores related to outcomes.
(2) Multimodal assessments added to LUS. Modalities of different evaluation (imaging radiography, computerized tomography, bioelectrical impedance analysis (BIA), laboratory biomarkers, and echocardiographic parameters) in heart failure diagnosis and follow-up.
(3) Lung ultrasound as therapeutic guide to assessing lung congestion in out-patient settings.

### 2.3. Data Extraction and Analysis

The data were extracted by two researchers independently in a standardized way, and then cross-checked the results. If there was disagreement, a discussion or decision by a third party was conducted. The primary extracted contents include the first author, publication year, objectives, study design, methodology (participants and instruments), outcomes, and main results.

### 2.4. Quality Assessment

Quality assessment was performed using the Newcastle-Ottawa Scale [42]. Publication bias was assessed using the Deeks funnel plot asymmetry test to determine its presence. A significance level of *p* < 0.05 was considered statistically significant.

## 3. Results

Figure 1 illustrates the search strategy employed. The present review included a total of 32 articles: 19 focused on the follow-up and prognostic assessment of heart failure, 11 centered on multimodal diagnostic assessments, and two addressing therapeutic guidance for heart failure diagnosis in the out-patient setting, encompassing diagnostic, therapeutic, and prognostic defined values.

Table 1 includes the chosen articles focusing on the precision of LUS as a diagnostic tool as well as the prognostic criteria of readmission and/or all-cause death after hospital discharge of patients admitted due to an episode of decompensated heart failure and/or ambulatory following-up:

(1) Diagnostic Accuracy in Heart Failure (HF) Suspicion [43,44]: LUS examinations were conducted on specific thoracic areas, including two anterior (A), two lateral (L), and two posterior (P) areas per hemithorax. An area was considered positive if three or more B-lines were observed. Diagnostic accuracy was determined by the number of positive areas identified: two positive areas out of four (Anterior-Lateral) on each hemithorax and two positive areas out of six (A-L-P) on each hemithorax. It was showed that incorporating LUS results may enhance the predictive capability of contemporary HF risk scores [45]. However, the impact of repeated ultrasound scans on prognostic outcomes remains uncertain [46].
(2) LUS in Stable Chronic HF Patients: LUS was effective in identifying stable chronic HF patients at high risk of death or HF hospitalization. At discharge, approximately 48.2% of patients exhibited a normal LUS profile [47,48]. The prognostic significance of the number of B-lines varied across studies. Most studies indicated the presence of ≥5 B-lines was found to be associated with a higher probability of 12-month all-cause death, while the presence of ≥15 B-lines was associated with a higher probability of HF readmission [44,48,49,50,51,52,53,54]. Others [54] suggested that the accumulation of 30–40 B-lines upon admission was identified as a risk factor for readmission or mortality, and the presence of ≥15 B-lines could just indicate an increased risk of persistent pulmonary congestion. Each additional B-line was associated with a 1.82 odds ratio for adverse outcomes [47], or a 3–4% increased risk for each additional B-line, as per reference [50].
(3) LUS-Guided Treatment: LUS-guided treatment was linked to a 45% reduction in the risk of hospitalization and a decrease in urgent visits [45,46,55,56,57] with follow-up after three months, six months, up to one year. However, no significant differences in death rates were observed [55,56,57]. Additionally, treatment guided by lung ultrasound (LUS) was linked to a reduced risk of Major Adverse Cardiac Events (MACEs) [58,59], and a significantly greater reduction in the number of B-lines during the initial 48 h, but it did not reduce heart failure readmission [57,60,61].
(4) The results of LUS remained independent of NT-proBNP levels [32,43,50,62]. It seems there is not any statistically significant association between median NT-proBNP levels among patients with a positive LUS for congestion and basal median NT-proBNP levels in patients with LUS without signs of congestion.

**Table 1:** B-lines and Heart Failure vs. outcomes.

First Author Year of Publication Country	Objectives	Methods				Results
		Design	Participants	Instruments, Procedure	Outcomes	
Platz E et al. 2019 [47] (EEUU)	To assess the prevalence, changes in, and prognostic importance of B-lines	Prospective, observational study	N = 349	4-zone LUS was performed at discharge. B-lines were quantified off-line, blinded to clinical findings and outcomes.	Risk of HF hospitalization or all-cause death	The OR ratio for each B-line was 1.82 (95% CI 1.14 to 2.88; *p* = 0.011) after adjusting for important clinical variables.
Kobalava Zh D et al. 2019 [49] (Russia)	To assess the prognostic significance of B-lines number at discharge.	Observational descriptive	N = 162	B lines at hospital discharge	Probability of 12-month all-cause death and probability of HF readmission.	At discharge normal LUS profile was observed in 48.2% of patients. Sum of B-lines ≥ 5 was associated with higher probability of 12-month all-cause death ([HR] 2.86, 95% CI 1.15–7.13, *p* = 0.024); and B-lines ≥ 15 B-lines with higher probability of HF readmission (HR 2.83, 95% CI 1.41–5.67, *p* = 0.003).
Marini et al., 2020 [55] (Italy)	To evaluate the usefulness of LUS + physical examination (PE) in the management of out-patients with acute decompensated heart failure (ADHF).	Randomized, multicenter, and unblinded study	N = 244	PE + LUS’ group vs. ‘PE only’ group.	Hospitalization rate for ADHF at 90-day follow-up.	The hospitalization was significantly reduced in ‘PE + LUS’ group with a reduction of risk for hospitalization by 56% (*p* = 0.01). There were no differences in mortality between the two groups.
Araiza-Garaigordobil et al., 2020 [56] (Mexico)	LUS during follow-up of patients with HF may reduce the rate of adverse events compared with usual care.	Randomized, single-center, blinded, and controlled trial CLUSTER-HF study	N = 126	LUS vs. usual care	Urgent visits, rehospitalization for worsening HF, and death from any cause during a 6-month period.	LUS-guided treatment was associated with a 45% risk reduction for hospitalization (HR 0.55, 95% CI 0.31–0.98, *p* = 0.044), and reduction in urgent visits (HR 0.28, 95% CI 0.13–0.62, *p* = 0.001). No significant differences in death were found.
Rivas-Lasarte M et al., 2019 [62] (Spain)	To evaluate relationship between results LUS-guided follow-up protocol and reduction NT-proBNP.	Randomized, single-blind clinical trial.	N = 123	A standard follow-up (n = 62, control group) or a LUS-guided follow-up (n = 61, LUS group)	urgent visit, hospitalization and death, at 14, 30, 90 and 180 days after discharge	Reduction the number of decompensations and improved walking capacity, but N-terminal pro-B-type natriuretic peptide reduction were not achieved.
Conangla et al., 2020 [43] (Spain)	LUS improved diagnostic accuracy in HF suspicion.	Prospective study of LUS in ambulatory patients > 50 years old	N = 223	LUS was performed on 2 anterior (A), 2 lateral (L), and 2 posterior (P) areas per hemithorax. An area was positive when ≥3 B-lines were observed.	Two diagnostic criteria were used: for LUS-C1, 2 positive areas of 4 (A-L) on each hemithorax; and for LUS-C2, 2 positive areas of 6 (A-L-P) on each hemithorax.	LUS was accurate enough to rule-in HF in a primary care setting irrespective NT-proBNP availability.
Domingo M, et al. 2021 [50] (Spain)	The prognostic value of LUS.	Observational, prospective, single-center cohort study	N = 577	LUS was performed in situ. The sum of B-lines across all lung zones and the quartiles of this addition were used for the analyses.	The main clinical outcomes were a composite of all-cause death or hospitalization for HF and mortality from any cause during mean follow-up of 31 ± 7 months.	The mean number of B-lines was 5 ± 6. Having ≥ 8 B-lines doubled the risk of the composite primary event (*p* < 0.001) and increased the risk of death from any cause by 2.6-fold (*p* < 0.001) with a 3% to 4% increased risk for each 1-line addition irrespective NT-proBNP level.
Wang Y et al., 2021 [51] (Brasil)	Prognostic value of lung ultrasound assessed by B-lines	A Systematic Review and Meta-Analysis	Nine studies involving N = 1212	HF out-patients	Outcomes of all-cause mortality or HF hospitalization	B-lines > 15 and >30 at discharge were significantly associated with increased risk of combined outcomes
Rueda-Camino JA et al. 2021 [52] (Spain)	To determine the diagnostic accuracy of bedside LUS prognostic tool for HF suspicion	Prospective cohort study		B lines: two groups were formed: less than 15 B-lines (unexposed) and ≥15 B-lines (exposed).	Risk of readmission and mortality with 3-month follow-up	Patients with ≥15 B-lines are 2.5 times more likely to be readmitted (HR: 2.39; 95%CI: 1.12–5.12; *p* = 0.024), without differences in terms of mortality.
Zisis G et al., 2022 [60] (Australia)	To evaluate the efficacy a nurse-led, LUICA-guided disease management program (DMP)	RISK-HF randomized controlled trial	N = 404	Patients at high risk for 30-day readmission and/or death to LUS-guided DMP or usual care.	LUS was performed at discharge and at least twice in the first month of follow-up	Handheld ultrasound at and after hospital discharge improves fluid status but does not reduce heart failure readmission.
Maestro-Benedicto, A et al., 2022 [45] (Spain)	contemporary HF risk scores can be improved upon by the inclusion of the number of B-lines detected by LUS	Randomized, single-center, simple blind trial	N = 123	LUS at discharge contemporary HF risk scores at 15 days, 1, 3 and 6 months after the hospitalization	predict death, urgent visit, or HF readmission at 6-month	Adding the results of LUS evaluated at discharge improved the predictive value of most of the contemporary HF risk scores in the 1-month score and 1-year.
Mhanna M et al., 2022 [57] (EEUU)	A point-of-care lung ultrasound (LUS) is a useful tool to detect subclinical pulmonary edema.	Systematic review and meta-analysis	N = 493	LUS plus PE-guided therapy vs. managed with PE-guided therapy alone	HF hospitalization, all-cause mortality, urgent visits for HF worsening, acute kidney injury (AKI), and hypokalemia rates.	Out-patient LUS-guided diuretic therapy of pulmonary congestion reduces urgent visits for worsening symptoms of HF. No significant difference in HF hospitalization rate. Similarly, there was no significant difference in all-cause mortality, and hypokalemia.
Rattarasan I et al., 2022 [48] (Thailand)	Evaluate the prognostic value of B-lines for prediction of rehospitalization and death	Prospective cohort	N = 126	B-lines and the size of the inferior vena cava. Two groups were formed: B-lines (<12) vs. B-lines (≥12)	Prediction of readmission hospitalization and death within 6 months	The mean number of B-lines at discharge was 9 ± 9, and the presence ≥ 12 B-lines before discharge was an independent predictor of events at 6 months
Dubon-Peralta E et al. 2022 [54] (Spain)	assessment of pulmonary congestion in patients with heart failure	A systematic review	14 articles	evaluate the prognostic significance of the presence of B lines detected by LUS	Optimization of treatment by monitoring the dynamic changes	The presence of more than 30–40 B lines at admission were considered a risk factor for readmission or mortality as was persistent pulmonary congestion with the presence of ≥15 B-lines.
Arvig MD et al., 2022 [46] (Denmark)	investigate if treatment guided by serial LUS compared to standard monitoring	Systematic search	24 studies N = 2040	serial LUS of the inferior vena cava-collapsibility index (IVC-CI) and B-lines on LUS	mortality, readmissions	A single ultrasound measurement can influence prognostic outcomes, but it remains uncertain if repeated scans can have the same impact.
Yan Li et al., 2022 [58] (China)	to evaluate the usefulness of LUS-guided treatment vs. usual care in reducing the major adverse cardiac event (MACE) rate	systematic review and meta-analysis of randomized controlled trials	10 studies N = 1203	LUS-guided treatment vs. usual care a, LUS-guided treatment	MACEs, all-cause mortality, and HF-related rehospitalization, during mean follow-up of 4.7 months	The meta-regression analysis showed a significant correlation between MACEs and the change in B-line count (*p* < 0.05). LUS-guided treatment was associated with a significantly lower risk of MACEs.
Platz E et al., 2023 [59] (EEUU)	PARADISE-MI Assess the trajectory of pulmonary congestion using lung ultrasound (LUS)	Prospective cohort study	N = 152	LUS underwent 8-zone LUS and echocardiography at baseline (±2 days of randomization) and after 8 months.	Patients with acute myocardial Left ventricular ejection fraction, pulmonary congestion or both	The proportion of patients without pulmonary congestion at follow-up was significantly higher in those with fewer B-lines at baseline
Cohen et al., 2023 [44] (EEUU)	Association between numbers of B-lines on LUS.	Prospective study of adults	200 patients at discharge	Number of B-lines. By an 8-zone LUS exam to evaluate for the presence of B-lines	Risk of 30-day readmission in patients hospitalized for acute decompensated HF.	The presence of B-lines at discharge was associated with a significantly increased risk of 30-day readmission. Compared with patients with 0–1 positive zones, patients with 2–3 positive lung zones was 1.25 times higher (95% CI: 1.08–1.45), and with 4–8 positive lung zones was 1.50 times higher (95% CI: 1.23–1.82).
Goldsmith AJ et al., 2023 [61] (EEUU)	BLUSHED-AHF study: to explore whether LUS early targeted intervention vs. leads improves pulmonary congestion	Multicenter, randomized, pilot trial	N = 130	LUS-guided protocol	Number of B-lines at 6 h or in 30 days	LUS conferred no benefit compared with usual care in reducing the number of B-lines at 6 h or in 30 days, but a significantly greater reduction in the number of B-lines was observed in LUS-guided patients during the first 48 h.

Table 2 encompasses selected articles concentrating on the LUS in conjunction with other commonly used assessments. These include clinical assessment versus chest radiography with or without inferior vena cava (IVC) ultrasound, LUS versus computerized tomography (CT), Rx thoracic, bioelectrical impedance analysis (BIA), early diagnosis through exercise LUS, laboratory parameters (pro-BNP, CA125), and echocardiographic parameters.

The LUS showed higher sensitivity ratio 1.2 (95% CI, 1.08–1.34; *p* < 0.001) compared with CxR, computerized tomography (CT), and echocardiogram [61,62,63,64,65,66] in the diagnosis of HF and using LUS with the clinical evaluation reduced diagnostic errors as compared to [CxR + Nt-proBNP] combination [67]. However, mortality was significantly associated with lower inferior vena cava (IVC) collapse [53,65,68] and a higher number of lung B-lines, as well as elevated NT-proBNP levels [54,68,69,70], with no differences observed in the bioelectrical impedance analysis (BIA) parameters. Among the majority of individuals with ambulatory follow-up and preserved ejection fraction [71,72], the submaximal exercise increases B-lines number to level of higher probability of 12-month all-cause death or/and higher probability of HF decompensation [44,48,49,50,51,52]. The total sum of B-lines correlated significantly, but fairly, with congestion and several biomarkers, especially with high-sensitive Troponin T (hsTnT) [73].

**Table 2:** Multimodal assessment (clinical, laboratory, and LUS).

First Author Year of Publication Country	Objectives	Methods				Results
		Design	Participants	Instruments	Outcomes	
Maw AM et al., 2019 [63] (EEUU)	To compare the accuracy of LUS with the accuracy of chest radiography (CxR) in the diagnosis of HF.	Systematic Review and Meta-analysis Prospective cohorts	6 studies N = 1827	LUS vs. CxR	Detection of cardiogenic pulmonary edema	Sensitivity LUS vs. CxR 0.88 (95% Cl, 0.75–0.95) vs. 0.73 (95% CI, 0.70–0.76) Specificity LUS vs. CxR 0.90 (95% Cl, 0.88–0.92) vs. 0.90 (95% CI, 0.75–0.97).
Pivetta E et al., 2019 [67] (Italy)	To evaluate accuracy of combining [LUS] vs. [CxR + NT-proBNP]	Randomized trial	N = 518	Either LUS or [CXR/NT + proBNP]	HF diagnosis accuracy	LUS was higher than [CXR/Nt-proBNP] (AUC 0.95 vs. 0.87, *p* < 0.01).
Curbelo et al., 2019 [54] (Spain)	Comparing the usefulness of inferior vena cava (IVC) ultrasound, lung ultrasound, bioelectrical impedance analysis (BIA), and (NT-proBNP)	Prospective cohort study	N = 99	LUS IVC BIA NT-proBNP	Parameters of congestion and mortality	Mortality was associated to significantly lower IVC collapse, and a greater number of lung B-lines; and higher NTproBNP levels. No differences in the BIA parameters.
Reddy V et al., 2019 [71] (EEUU)	To evaluate increases in Extravascular water at rest and during exercise	Observacional	N = 66	LUS during invasive hemodynamic submaximal exercise testing	B-lines increase during exercise	54% (n = 33) either developed new B-lines (n = 23, 38%) or developed an increase in the number B-lines (n = 10, 16%) during exercise.
Domingo M et al. 2020 [73] (Spain)	To assess relationship between B-lines assessed by LUS and biomarkers	prospective cohort of ambulatory patients	N = 170	12-scan LUS protocol (8 anterolateral areas plus 4 lower posterior thoracic areas) and 11 inflammatory and cardiovascular biomarkers	confirmed HF diagnosis	total B-line sum significantly correlated with NT-proBNP (R = 0.29, *p* < 0.001), growth/differentiation factor-15 (GDF-15; R = 0.23, *p* = 0.003), high-sensitive Troponin T (hsTnT; R = 0.36, *p* < 0.001), soluble interleukin-1 receptor-like 1 (sST2; R = 0.29, *p* < 0.001), cancer antigen 125 (CA-125; R = 0.17, *p* = 0.03), high-sensitivity C-reactive protein (hsCRP; R = 0.20, *p* = 0.009), and interleukin (IL)-6 (R = 0.23, *p* = 0.003).
Rubio-Gracia J et al., 2021 [69] (Spain)	Evaluate LUS associated to NT-proBNP, cancer antigen 125, relative plasma volume (rPV) estimation.	Retrospective study	N = 203	LUS CA 125 NT-proBNP rPV	Parameters of venous congestion and predictors of mortality after one year of follow-up.	Values of NT-proBNP ≥ 3804 pg/mL (HR 2.78 [1.27–6.08]; *p* = 0.010) and rPV ≥ −4.54% (HR 2.74 [1.18–6.38]; *p* = 0.019) were independent predictors of all-cause mortality
Morvai-Illés B et al., 2021 [70] (Hungary)	LUS B-lines compared vs. echocardiographic parameters and natriuretic peptide level	prospective cohort study	N = 75	B-lines LUS NT-proBNP	The prognostic value of B-lines and other novel ultrasound parameters: global longitudinal strain and left atrial reservoir strain.	≥15 B-lines lines was associated with a significantly worse event-free survival, and was similar to the predictive value of NT-proBNP (AUC 0.863 vs. 0.859)
Burgos et al. 2022 [68] (Argentina)	To evaluate if inferior vena cava (IVC) and lung ultrasound (CAVAL US)-guided therapy.	CAVAL US-AHF Study- Randomized control trial	N = 58	Assigned either LUS + IVC (‘intervention group’) or clinical-guided decongestion therapy (‘control group’), B-lines IVC readmission	Presence ≥ 5 B-lines and/or an increase in the diameter of the IVC, with and without collapsibility. Endpoints: the composite of readmission for HF, unplanned visit for worsening HF, variation of NT-proBNP or death at 90 days.	Mortality was associated to significantly lower IVC collapse, and a greater number of lung B-lines; and higher NTproBNP levels B-lines at discharge was associated with a significantly increased risk of 30-day readmission
Pérez-Herrero S et al., 2022 [65] (Spain)	To compare the CxR vs. B-lines by LUS and collapsibility of IVC.	Observational cohort study based on data collected in the PROFUND-IC study.	N = 301	CxR B-lines by LUS IVC	prediction of 30-day mortality based on the diameter of the IVC	≥6 B-lines per field and IVC collapsibility ≤ 50% had higher 30-day mortality rates
Chiu L et al., 2022 [64] (EEUU)	LUS diagnostic accuracy vs. a chest X-ray (CxR)	Meta-Analysis	8 studies N = 2787	LUS vs. chest radiography	diagnostic accuracy HF	LUS is more sensitive (91.8% vs. 76.5%) and more specific than CxR (92.3% vs. 87.0%) than CXR in detecting pulmonary edema.
Coiro S et al., 2023 [72] (France) [72]	Assess the diagnosis value of exercise lung ultrasound (LUS) for HF with preserved ejection fraction (HFpEF) diagnosis.	Case-control study	N = 116	B-line kinetics in submaximal exercise	Peak B-lines for HFpEF diagnosis	Peak B-lines > 5 were the best cutoffs for HFpEF diagnosis
Xie C et al., 2023 [66] (Xina)	LUS accuracy vs. computerized tomography (CT) vs. echocardiogram	Systematic review and Metanalysis	N = 345	LUS, (CT), and conventional echocardiogram	predictive value for HF diagnosis	The accuracy of LUS was significantly higher than that of echocardiogram (*p* = 0.01).

Table 3 includes a residual section featuring articles on LUS and therapeutic guidance based on the presence of lung ultrasound signs of congestion in ambulatory patients. Few studies have been found regarding the utility of Lung Ultrasound (LUS) as a guide for heart failure (HF) treatment in the context of out-patient follow-up, and the results obtained do not support the use of LUS in relation to its mortality and/or hospital readmission outcomes [74]. Although in clinical practice there was a higher likelihood of modifying diuretic treatment based on Lung Ultrasound (LUS) results, no differences were observed in the incidence of adverse events related to heart failure (HF) [74,75].

## 4. Discussion

This systematic review was conducted with the objective of identifying scientific evidence pertaining to the application of lung ultrasound in Heart Failure. Despite encountering a substantial number of trials lacking posted results, the review exhibits notable advantages in the detection of sub-clinical congestion linked to prognostic significance in terms of re-hospitalization, as indicated by the prognostic value associated with B-lines, and mortality prediction, both of which constitute pivotal attributes of heart failure in out-patient settings. The enhanced accuracy, surpassing that of physical examination and chest X-ray by 90%, establishes LUS as a superior diagnostic modality for congestion, and facilitates expedited diagnoses in the emergency department. Additionally, it confers incremental prognostic value during the hospital discharge phase of patients experiencing decompensated HF, and it may play a pivotal role in guiding the treatment strategies for individuals with HF.

However, despite the extensive evidence supporting the use of LUS across various medical disciplines, there is a notable scarcity of information regarding its application and interpretation criteria in the out-patient monitoring of HF patients within primary care settings. This knowledge gap persists despite the significant healthcare and economic challenges posed by the aging demographics of society. Recognizing the evolving landscape, a multidisciplinary and international panel LUS experts undertook a thorough review and update of the original international consensus on point-of-care LUS, initially established in 2012 [76,77]. Also, a statement has been published aimed at pulmonologists utilizing thoracic ultrasound within the realm of respiratory medicine [78]. The updated consensus reflects the advancements in LUS technology and its applications, providing a contemporary framework for practitioners. Ultimately, a clinical consensus statement of the European Association of Cardiovascular Imaging [79] becomes the reference standard in heart failure care and facilitates the exclusion of other highly prevalent conditions that may mimic or overlap with HF. Despite these strides, there remains a need for further research and exploration, particularly in the context of out-patient monitoring of HF patients in primary care.

In fact, international clinical practice guidelines on heart failure do not include standardized interpretation criteria for the predictive value of B-lines associated with intervention patterns, differential diagnosis, and the potential benefit of its use in terms of cost-effectiveness. Despite a recommendation (Class I, level B) by the ESC Guidelines [14,24], which suggests an intensive strategy involving the initiation and rapid up-titration of evidence-based treatment before discharge, along with frequent and careful follow-up visits in the first 6 weeks following heart failure hospitalization to reduce the risk of HF rehospitalization or all-cause death at 180 days, it does not specifically advocate the use of lung ultrasound for detecting pulmonary congestion in out-patients with heart failure. Furthermore, approximately 40% of patients are discharged prematurely from HF hospitalization when they may not be adequately prepared for discharge [20].

In the studies that were included, although they present similar results, there are some limitations such as their interpretation, highlighting the challenges in establishing their utility as valuable and non-invasive tools for monitoring changes in pulmonary congestion. These limitations include the absence of studies conducted in comparable populations and the inconsistent reporting of the technique employed and variable quantification of ‘B-lines’ jeopardize the reproducibility of LUS studies. Additionally, there is diversity in the healthcare areas from which the results originate, the dynamic nature of LUS findings in response to therapy, and differences in severity of disease complexity between healthcare levels. This includes interpretability challenges, especially between hospital care and out-patient follow-up in primary care.

Notably, the persistence of residual congestion at the time of hospital discharge serves as an indicator for individuals at heightened risk for adverse events [43,47,50]. On the other hand, the observation that in most individuals undergoing ambulatory follow-up with preserved ejection fraction [26,33], submaximal exercise increases the number of B-lines to a level associated with a higher probability of 12-month all-cause death and/or a higher likelihood of heart failure decompensation [2,9,11,15,20] may be an uncertain finding in terms of its prognostic value and implications for treatment. Recognizing the importance of early diagnosis, particularly in light of its contribution to the prompt implementation of appropriate treatment, is crucial for mitigating heart failure mortality. Finally, there is increased controversy concerning the effectiveness and correlation of changes in the B-lines pattern with monitoring congestion during optimizing therapy, serving as a guide in the outcomes and use of diuretics. Diuretics are considered one of the more affordable treatment approaches in the primary care setting. The need for an updated international consensus on the use of LUS is obvious [80].

Therefore, there are several potential benefits of using LUS to monitor heart failure patients in primary care:

- LUS can detect changes in lung function in heart failure patients before they become clinically apparent. Several studies have correlated the presence of B-lines on LUS with a sensitive marker for diagnosing decompensated HF. Residual pulmonary congestion at discharge, indicated by a B-line count ≥30, serves as a strong predictor of outcomes. However, in an HF out-patient clinic, a B-line count ≥15 cut-off could be considered for a rapid and reliable assessment of decompensation in HF out-patients [41,80]. This early detection can help clinicians intervene earlier, potentially reducing the severity of the patient’s condition and preventing the need for hospitalization. It should not be considered a substitute for imaging technology but rather a complementary tool in emergency and out-patient assessments.
- The implementation of lung ultrasound in primary care not only facilitates early detection of changes in lung function and improves patient outcomes but also promotes increased patient engagement [39,40,72]. This patient-centric approach, coupled with the non-invasive and cost-effective nature of lung ultrasound, represents a promising avenue for enhancing the overall quality of care for heart failure patients in primary care settings.
- As the population ages globally, there is a simultaneous increase in the prevalence of multiple comorbidities. The convergence of these demographic and health trends poses unique challenges for the healthcare system. It is becoming increasingly essential to address the healthcare needs of older individuals who may have complex medical conditions and varying degrees of mobility. The LUS has an incremental value in follow-up, the diagnostic and prognostic approach in potential complex scenarios as the bedside in non-traditional healthcare settings such as patients’ homes or institutional long-term care facilities. The early detection capabilities of LUS empower clinicians to intervene at an earlier stage, potentially mitigating the severity of the patient’s condition and averting the need for readmission [1,81]. This proactive approach to post-hospitalization care aligns with the goals of improving patient outcomes and reducing the burden on healthcare resources.
- Non-invasive and cost-effective: Lung ultrasound is a non-invasive and cost-effective method of monitoring heart failure patients. Unlike other diagnostic tests, such as CT scans, it does not expose patients to radiation and is more affordable [1,79]. While echocardiography plays a pivotal role in evaluating underlying cardiac structure and function, its effectiveness is highly dependent on the experience of the sonographer for image acquisition and precise interpretation by an expert reader. As a result, several Machine Learning-based platforms are being developed.
- The studies found that lung ultrasound was more accurate than clinical assessment, natriuretic peptides, and echo-Doppler cardiac parameters for detecting pulmonary congestion. Additionally, patients who received lung ultrasound as part of their care had a lower risk of death and hospitalization than those who did not [63,72,79]. Additionally, weak or moderate [24] correlations were found between serum biomarkers and LUS scores.
- As well as a pharmacologic therapeutic guide, LUS is also used in other clinical areas such as out-patient, pre- and per-operative, hemodialysis, septic shock, cardiogenic shock, teach-back educational, and pediatric care.

Moreover, the development of new research about LUS in various objectives must be considered, including: 1/integrating LUS technology into primary care to monitor out-patients at high and moderate risk of hospitalization or mortality, and post-hospital discharge; 2/creating a predictive model that utilizes a risk score and/or Artificial Intelligence; 3/assessing the accuracy and performance of the predictive model in a cost-effective manner using a validation dataset; 4/identifying high-risk subgroups within the patient population 5/exploring gender-associated differences in the context of heart failure; 6/coordinating efforts between primary care and the Heart Failure Unit for the comprehensive follow-up of heart failure patients. It is a main limitation that LUS is neither systematically incorporated as part of routine hospital discharge, nor as clinical activities in primary care in follow-up and early diagnosis of decompensation.

## 5. Conclusions

Lung ultrasound offers advantages in detecting subclinical congestion, which holds prognostic significance for rehospitalization and mortality prediction in heart failure patients, guiding treatment strategies effectively. In primary care, LUS provides benefit during hospital discharge and serves as a valuable, non-invasive tool for monitoring pulmonary congestion changes in various healthcare settings, including patients’ homes and long-term care facilities. However, there are uncertainties in interpreting findings, highlighting the need for an updated international consensus on LUS utilization in HF management.

## Figures and Tables

**Table 3:** Therapeutic guidance based on the presence of lung ultrasound signs of congestion in ambulatory patients.

First Author Year of Publication Country	Objectives	Methods				Results
		Design	Participants	Instruments	Outcomes	
Torres-Macho J et al., 2022 [74] (Spain)	to evaluate if LUS-guided diuretic therapy could improve short- and mid-term prognosis compared with standard of care (SOC) after discharge	Randomized, multicentre, single-blind clinical trial (EPICC trial)	N = 79	Participants will be assigned 1:1 to receive treatment guided according to LUS signs of congestion (semi-quantitative evaluation of B lines and the presence of pleural effusion) vs. SOC.	Combination of cardiovascular death and readmission for HF at 6 months.	LUS did not show any benefit in survival analysis or a need for intravenous diuretics compared with SOC.
Cruz M et al. 2023 [75] (Portugal)	LUS results to the HF assistant physician would change loop diuretic adjustments in “stable” chronic ambulatory HF patients.	Prospective randomised single-blinded trial	N = 139	70 were randomised to blind LUS and 69 to open LUS.	The primary outcome was change in loop diuretic dose (up- or down-titration).	Clinicians were more likely to titrate furosemide dose, but the risk of HF events or cardiovascular death did not differ.
